# Immunohistochemical markers as predictors of prognosis in multifocal prostate cancer

**DOI:** 10.1007/s00428-023-03699-z

**Published:** 2023-11-28

**Authors:** Laura Segalés, Nuria Juanpere, Nerea Gallarín, Marta Lorenzo, David López, Júlia Perera-Bel, Alejo Rodriguez-Vida, Lluís Fumadó, Lluís Cecchini, Joaquim Bellmunt, Josep Lloreta-Trull, Silvia Hernández-Llodrà

**Affiliations:** 1 Department of Medicine and Life Sciences, Universitat Pompeu Fabra, Barcelona, Spain; 2 Department of Pathology, Hospital del Mar, Barcelona, Spain; 3 Hospital del Mar Research Institute, Barcelona, Spain; 4 Department of Medical Oncology, Hospital del Mar, CIBERONC, Barcelona, Spain; 5 Department of Urology, Hospital del Mar, Barcelona, Spain; 6 Dana Farber Cancer Institute, Harvard Medical School, Boston, MA USA

**Keywords:** Prostate cancer, Immunohistochemical panel, Prognosis, Focality, Heterogeneity

## Abstract

The impact of tumor focality on prostate cancer (PCa) prognosis has been addressed in several studies with conflicting results. Tumor foci from multifocal (MF) PCa can show highly heterogeneous molecular features. Our aim was to analyze the protein expression of PTEN, SPOP, SLC45A3, ETV1, ERG and the “triple hit” (ERG overexpression, PTEN plus SLC45A3 loss) in unifocal (UF) and MF PCa, to evaluate their value as prognostic markers according to focality, and the role of tumor heterogeneity in MF disease. PTEN, SPOP, SLC45A3, ETV1 and ERG immunohistochemical expression was evaluated in 185 PCa from 9 TMAs, 51 UF and 134 MF. In a subset of 69 MF cases, the dominant and secondary foci (DF and SF) were compared. Heterogeneity was considered when both tumor foci presented different expression patterns. Relationship with clinicopathological features was also analyzed. MF PCa was diagnosed in significantly younger patients when compared to UF ones (*p* = 0.007). ETV1 overexpression was associated with UF disease (*p* = 0.028). A shorter time to PSA recurrence was related to SLC45A3 *wt* expression in UF PCa (*p* = 0.052), and to SPOP expression loss (*p* = 0.043) or “triple hit” phenotype in MF PCa (*p* = 0.041). In MF cases, PTEN loss, SLC45A3 loss and “triple hit” phenotype were associated with the DF and had significant heterogeneity. In conclusion, our results indicate that UF and MF PCa have relevant and consistent molecular differences. The analysis of an immunohistochemical panel, composed by PTEN, SPOP, SLC45A3, ETV1 and ERG, could be useful to predict outcome in MF cases.

**Supplementary Information:**

The online version contains supplementary material available at 10.1007/s00428-023-03699-z.

## Introduction

Prostate Cancer (PCa) is a highly heterogeneous disease from the clinical, anatomical and molecular points of view [1, 2]. Its clinical course differs vastly and it often presents as a multifocal (MF) malignancy [3]. Some studies have reported multiple tumor foci in 60% to 90% of cases [1, 4–10]. Molecular features can be so variable among tumor areas in a given MF PCa as they can be among unifocal (UF) PCa from different patients [1, 5]. Several studies have shown that low- and high-grade tumor foci in MF PCa exhibit different molecular signatures, including tumor mutational burden, copy number alterations, gene expression profiles, weighted genome instability index, proteomics and clonal evolutionary processes [11–15]. Different theories have tried to explain the origin of heterogeneity in MF disease. In cases with multiclonal origin, each focus would evolve independently through the accumulation of different alterations. Alternatively, an initially uniclonal tumor could give rise to multiple tumor foci by intraglandular dissemination plus accumulation of alterations over time [6, 16, 17]. In fact, both models are possible, and they can even be found combined in some cases [16, 18].

The Cancer Genome Atlas reported that, based on molecular alterations, 74% of clinically-localized PCa can be classified in two major carcinogenic pathways: one related to *ETS* fusions and another related to somatic mutations [19]. In both pathways, frequent copy number alterations have been considered potential drivers of progression [20]. In previous studies, we analyzed the protein expression of relevant genes from the two major PCa pathways, mainly *PTEN*, *SPOP*, *SLC45A3*, *ETV1* and *ERG*, as well as the “triple hit” combination (*ERG* overexpression, *PTEN* plus *SLC45A3* loss*)*, and we reported relevant associations between changes in these molecules and the clinicopathological features of the tumors [21–23].

In the present study, we aimed to understand the role of PTEN, SPOP, SLC45A3, ETV1 and ERG alterations in UF and MF PCa etiopathogenesis, to assess their value as prognostic markers, and the heterogeneity in the expression of these proteins in MF disease. With this purpose, we analyzed their immunohistochemical expression, individually or as components of the “triple hit” combination, in a well-defined series of UF and MF localized PCa.

## Materials and methods

### Patients and tumor samples

One hundred and eighty-five patients with PCa who underwent radical prostatectomy were retrospectively selected from the files of the Hospital del MAR Biobank (MARBiobanc, Barcelona, Spain). Grade Group (GG) or ISUP/WHO grade at diagnosis, age at diagnosis, pre-operative PSA levels, tumor stage, biochemical recurrence, perineural infiltration, extra-prostatic extension, seminal vesicle invasion, resection margin status, and tumor focality information was retrieved from the patients’ clinical history records, and it is shown in Table 1. Grade Group at diagnosis refers to the GG of the single tumor focus in UF cases, and the GG of the dominant focus in MF cases (the highest GG). Perineural infiltration was defined as the invasion of the extra-prostatic neurovascular bundles by the tumor. Tumor focality was classified as UF *vs* MF. Multifocality was considered when at least two tumor foci were identified in the prostatectomy specimens, without any overlap between them in the axial and sagittal planes of consecutive prostate sections.

**Table 1:** Summary of clinical data

GG at diagnosis		GG1, N = 29	GG2, N = 69	GG3, N = 21	GG4, N = 25	GG5, N = 41
Age at diagnosis, range (average)		52 to 75 (65)	47 to 84 (65)	51 to 72 (64.9)	52 to 71 (63)	56 to 83 (65.9)
Pre-operative PSA (ng/ml), range (average)		0.4 to 51 (7.6)	2.6 to 16.8 (7.1)	3.8 to 16.1 (7.1)	3.6 to 24.2 (9.4)	2.8 to 17.9 (7.9)
Tumor stage (pT), number of cases (%)	pT2, N = 130	24 (82.8%)	60 (87%)	14 (66.7%)	12 (48%)	20 (48.8%)
	pT3, N = 55	5 (17.2%)	9 (13%)	7 (33.3%)	13 (52%)	21 (51.2%)
PSA recurrence, number of cases (%)	Yes, N = 44	7 (25%)	15 (22.4%)	4 (19.1%)	6 (26.1%)	12 (30%)
	No, N = 135	21 (75%)	52 (77.6%)	17 (80.9%)	17 (73.9%)	28 (70%)
	N	N = 28	N = 67	N = 21	N = 23	N = 40
Perineurial infiltration, number of cases (%)	Yes, N = 43	3 (10.3%)	10 (14.5%)	5 (23.8%)	8 (32%)	17 (41.5%)
	No, N = 142	26 (89.7%)	59 (85.5%)	16 (76.2%)	17 (68%)	24 (58.5%)
Extra-prostatic extension, number of cases (%)	Yes, N = 86	8 (27.6%)	28 (40.6%)	11 (52.4%)	16 (64%)	23 (56.1%)
	No, N = 99	21 (72.4%)	41 (59.4%)	10 (47.6%)	9 (36%)	18 (43.9%)
Seminal vesicle invasion, number of cases (%)	Yes, N = 12	2 (6.9%)	0 (0%)	1 (4.8%)	2 (8%)	7 (17.1%)
	No, N = 171	27 (93.1%)	67 (100%)	20 (95.2%)	23 (92%)	34 (82.9%)
	N	N = 29	N = 67	N = 21	N = 25	N = 41
Resection margin status, number of cases (%)	Affected, N = 83	11 (37.9%)	26 (37.7%)	6 (28.6%)	12 (48%)	28 (68.3%)
	Unaffected, N = 102	18 (62.1%)	43 (62.3%)	15 (71.4%)	13 (52%)	13 (31.7%)
Tumor focality, number of cases (%)	Unifocal, N = 51	8 (27.6%)	15 (21.7%)	12 (57.1%)	5 (20%)	11 (26.8%)
	Multifocal, N = 134	21 (72.4%)	54 (78.3%)	9 (42.9%)	20 (80%)	30 (73.2%)

GG: Grade Group, pT: pathological tumor stage

### Immunohistochemistry

We selected 185 cases with complete PTEN, SPOP, SLC45A3, ETV1 and ERG immunostaining from a previous series of 230 PCa. From them, 51 were UF and 134 MF. Formalin-fixed paraffin-embedded PCa samples were included in 9 tissue microarrays (TMAs) [21–23]. All the cases were re-reviewed by two expert pathologists to confirm the grade of the PCa foci. In the UF cases, the single tumor focus was included. In 65 of the MF cases, only the dominant focus (DF, the one with the highest GG) was included. From the 69 remaining MF cases, at least two tumor foci were included in the TMAs. In the latter, two tumor foci have been considered, both the DF plus a secondary focus (SF, a focus with a lower GG than the DF).

As it has been previously described, PTEN, SPOP and SLC45A3 nuclear and cytoplasmic loss were assessed using a semi-quantitative scoring system considering two categories: *wt* or loss of expression. Adjacent normal tissue staining was used as an internal reference for PTEN and SLC45A3, and smooth muscle staining for SPOP [21, 23]. For ERG, *wt* or nuclear overexpression were considered [21]. Finally, ETV1 cytoplasmic expression was graded quantitatively by a histoscore system ([1 × (%1 + cells)] + [2 × (%2 + cells)] + [3 × (%3 + cells)]), and subsequently *wt* (0–99) and overexpression (≥ 100) categories were established [22]. For both ETV1 and ERG, endothelial cells were used as a positive internal control. Examples of altered and *wt* immunostainings are shown in Supplementary Fig. 1.

#### Statistical analysis

Categorical variables were presented as frequencies and percentages, and quantitative variables as average and ranges. Pearson Chi-Square, Fisher’s Exact or Wilcoxon Mann Whitney tests were used. Nominal *p*-values < 0.05 were considered statistically significant (not corrected for multiple testing). The McNemar test was used for the heterogeneity analysis, in which a *p-*value < 0.05 indicated significant heterogeneity between foci.

The relationship with time to PSA recurrence was analyzed by Cox proportional hazards regression and visualized using Kaplan–Meier curves. Log-Rank test was applied to compare the survival probability between groups in 179 patients (6 cases in this series were lost for follow-up). Multivariate Cox’s proportional hazards models were used to estimate adjusted hazard ratios (HR). Patients were followed at regular intervals of 3 months for one year and every 6 months for the subsequent years, and a PSA test was performed before every follow-up visit. None of the patients received pre- or post-operative radiation, nor adjuvant hormone therapy. Recurrence was defined as an increase in serum PSA > 0.2 ng/ml at the time of the last clinical follow-up appointment (i.e., two consecutive increases). Patients’ follow-up ranged from 5 to 274 months, with an average value of 92.8 months and a median of 96 months. In the PSA recurrence analysis, a *p*-value < 0.05 was considered statistically significant. Statistical analyses were performed using R programming language version 4.3.0 (R Foundation, Vienna, Austria).

## Results

### Clinicopathological characteristics of unifocal and multifocal prostate cancer

**Fig. 1:**
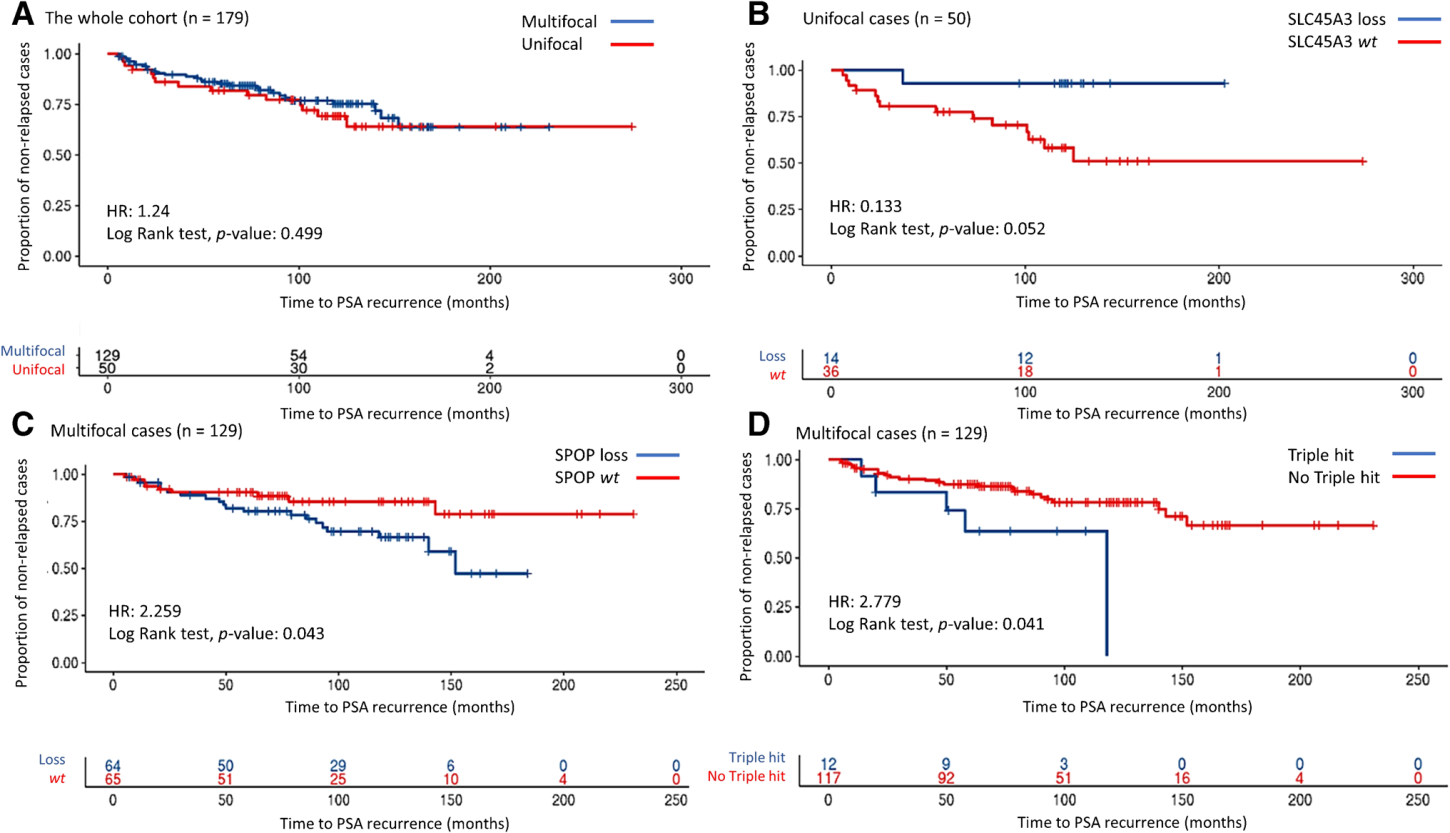
Kaplan Meier survival analysis for PSA recurrence in UF *vs* MF PCa (**a**), UF PCa with SLC45A3 loss *vs* SLC45A3 *wt* (**b**), MF PCa with SPOP loss *vs* SPOP *wt* (**c**) and MF PCa with *vs* without “triple hit” (**d**). *P*-values are obtained from Log Rank tests. UF: unifocal, MF: multifocal, PCa: prostate cancer

Tumors were classified as UF or MF, and no association was detected between most of the clinicopathological characteristics and tumor focality (Table 2a). However, MF cases were diagnosed in younger patients, as the average age at diagnosis was 64.2 years for men with MF and 66.9 years for men with UF tumors (*p* = 0.002). In addition, a different distribution of cases according to the PCa GG was observed in UF *vs* MF disease, especially in GG2 (29.4% *vs* 40.3%) and GG3 (23.5% *vs* 6.7%) groups (*p* = 0.025). Survival analysis was performed to compare the time to PSA recurrence in UF *vs* MF PCa, but no differences were detected (HR 1.24, *p* = 0.499) (Fig. 1a).

**Table 2:** Clinicopathological features (a) and immunohistochemical expression (b) in UF and MF PCa

A Clinicopathological characteristics		Unifocal tumors, N = 51	Multifocal tumors, N = 134	*p-value*
Age at diagnosis, range (average)		54 to 80 (66.9)	47 to 84 (64.2)	0.002 Ψ
GG at diagnosis, number of cases (%)	GG1, N = 29	8 (15.7%)	21 (15.7%)	0.025 *
	GG2, N = 69	15 (29.4%)	54 (40.3%)	
	GG3, N = 21	12 (23.5%)	9 (6.7%)	
	GG4, N = 25	5 (9.8%)	20 (14.9%)	
	GG5, N = 41	11 (21.6%)	30 (22.4%)	
Tumor stage (pT), number of cases (%)	pT2, N = 130	37 (72.5%)	93 (69.4%)	0.675 *
	pT3, N = 55	14 (27.5%)	41 (30.6%)	
Perineurial infiltration, number of cases (%)	Yes, N = 43	13 (25.5%)	30 (22.4%)	0.655 *
	No, N = 142	38 (74.5%)	104 (77.6%)	
Extra-prostatic extension, number of cases (%)	Yes, N = 86	27 (53%)	59 (44%)	0.277 *
	No, N = 99	24 (47%)	75 (56%)	
Seminal vesicle invasion, number of cases (%)	Yes, N = 12	5 (9.8%)	7 (5.3%)	0.320 Ω
	No, N = 171	46 (90.2%)	125 (94.7%)	
	N	N = 51	N = 132	
Resection margin status, number of cases (%)	Affected, N = 83	23 (45.1%)	60 (44.8%)	0.964 *
	Unaffected, N = 102	28 (54.9%)	74 (55.2%)	
B Immunohistochemical expression		Unifocal tumors, N = 51	Multifocal tumors, N = 134	*p-value*
PTEN, number of cases (%)	loss, N = 73	16 (31.4%)	57 (42.5%)	0.165 *
	*wt,* N = 112	35 (68.6%)	77 (57.5%)	
SPOP, number of cases (%)	loss, N = 93	27 (52.9%)	66 (49.3%)	0.654 *
	*wt,* N = 92	24 (47.1%)	68 (50.7%)	
SLC45A3, number of cases (%)	loss, N = 61	15 (29.4%)	46 (34.3%)	0.525 *
	*wt,* N = 124	36 (70.6%)	88 (65.7%)	
ETV1, number of cases (%)	overexpression, N = 64	24 (47.1%)	40 (29.8%)	0.028 *
	*wt,* N = 121	27 (52.9%)	94 (70.2%)	
ERG, number of cases (%)	overexpression, N = 95	27 (52.9%)	68 (50.7%)	0.789 *
	*wt,* N = 90	24 (47.1%)	66 (49.3%)	
ERG overexpression, PTEN and SLC45A3 loss, number of cases (%)	Triple hit, N = 18	5 (9.8%)	13 (9.7%)	0.983 *
	no Triple hit, N = 167	46 (90.2%)	121 (90.3%)	

P-values are obtained from Ψ Wilcoxon Mann Whitney, * Pearson Chi-Square or Ω Fisher’s Exact tests

### Immunohistochemical expression in unifocal and multifocal prostate cancer

Protein expression of PTEN, SPOP, SLC45A3, ETV1, ERG, and the so called “triple hit” [21, 24], were evaluated. Expression was considered as altered when loss or overexpression was present in the single focus from UF cases or at least in one of the tumor foci from MF ones. Expression alterations in UF and MF PCa were compared, and the results are shown in Table 2b. UF and MF tumors showed similar percentages of SPOP and SLC45A3 expression loss, ERG overexpression and “triple hit” phenotype. PTEN loss of expression was more frequent in MF cases, but without statistical significance. Interestingly, ETV1 overexpression was associated with UF disease (*p* = 0.028).

### Immunohistochemical expression and relationship with clinicopathological features according to focality

The relationship between immunohistochemical expression in UF and MF PCa and different clinicopathological features, such as age at diagnosis, GG at diagnosis, tumor stage, perineural infiltration, extra-prostatic extension, seminal vesicle invasion, and resection margin status was analyzed. In UF PCa, ETV1 overexpression was related to pT3 tumor stage (*p* = 0.031), and ERG overexpression was associated with perineural infiltration (*p* = 0.044), the latter also showing a trend to be related to younger patients (*p* = 0.058) (Supplementary Table 1).

In MF PCa, several clinicopathological features were strongly related to different protein expression alterations (Table 3). Thus, patients with MF PCa overexpressing ERG were younger (*p* = 0.069). Low GG was associated with *wt* expression of SPOP, SLC45A3 and ETV1 (*p* = 0.022; *p* = 0.025 and *p* = 0.010), as well as with the lack of the “triple hit” phenotype (*p* = 0.002), and showed a trend to be associated with PTEN *wt* (P = 0.059). Tumor stage pT3 was statistically related to PTEN and SPOP loss of expression (*p* = 0.012 and *p* = 0.011), as well as to ERG overexpression and the “triple hit” phenotype (*p* = 0.051 and *p* < 0.001). Perineural infiltration was also associated with PTEN loss (*p* = 0.002), ERG overexpression (*p* = 0.047) and the “triple hit” phenotype (*p* < 0.001). Finally, SPOP loss (P = 0.060) and the “triple hit” phenotype (*p* = 0.021) were detected in a high proportion of MF PCa with seminal vesicle invasion when compared to MF cases without this feature.

**Table 3:** Relationship between immunohistochemical expression and clinicopathological features in MF PCa

MULTIFOCAL PCa, N = 134		PTEN		SPOP		SLC45A3		ETV1		ERG		Triple hit	
		loss N = 57	*wt* N = 77	loss N = 66	*wt* N = 68	loss N = 46	*wt* N = 88	over- expression N = 40	*wt* N = 94	over- expression N = 68	*wt* N = 66	Yes N = 13	No N = 121
Age at diagnosis, range (average)		51 to 84 (63.6)	47 to 83 (64.6)	47 to 84 (63.7)	51 to 83 (64.7%)	47 to 75 (63.1)	51 to 84 (64.7)	51 to 83 (64.6)	47 to 84 (64)	47 to 75 (63.1)	52 to 84 (65.3)	54 to 75 (64.5)	47 to 84 (64.1)
*p-value*		0.290 Ψ		0.293 Ψ		0.306 Ψ		0.442 Ψ		0.069 Ψ		0.871 Ψ	
GG at diagnosis, number of cases (%)	GG1, N = 21	4 (19%)	17 (81%)	6 (28.6%)	15 (71.4%)	3 (14.3%)	18 (85.7%)	4 (19%)	17 (81%)	6 (28.6%)	15 (71.4%)	0 (0%)	21 (100%)
	GG2 + 3, N = 63	27 (42.9%)	36 (57.1%)	28 (44.4%)	35 (55.6%)	19 (30.2%)	44 (69.8%)	18 (28.6%)	45 (71.4%)	32 (50.8%)	31 (49.2%)	2 (3.2%)	61 (96.8%)
	GG4, N = 20	12 (60%)	8 (40%)	11 (55%)	9 (45%)	8 (40%)	12 (60%)	12 (60%)	8 (40%)	13 (65%)	7 (35%)	4 (20%)	16 (80%)
	GG5, N = 30	14 (46.7%)	16 (53.3%)	21 (70%)	9 (30%)	16 (53.3%)	14 (46.7%)	6 (20%)	24 (80%)	17 (56.7%)	13 (43.3%)	7 (23.3%)	23 (76.7%)
*p-value*		0.059 *		0.022 *		0.025 *		0.010 *		0.103 *		0.002 Ω	
Tumor stage (pT), number of cases (%)	pT2, N = 93	33 (35.5%)	60 (64.5%)	39 (41.9%)	54 (58.1%)	29 (31.2%)	64 (68.8%)	26 (28%)	67 (72%)	42 (45.2%)	51 (54.8%)	1 (1.1%)	92 (98.9%)
	pT3, N = 41	24 (58.5%)	17 (41.5%)	27 (65.9%)	14 (34.1%)	17 (41.5%)	24 (58.5%)	14 (34.1%)	27 (65.9%)	26 (63.4%)	15 (36.6%)	12 (29.3%)	29 (70.7%)
*p-value*		0.012 *		0.011 *		0.248 *		0.470 *		0.051 *		< 0.001 Ω	
Perineurial infiltration, number of cases (%)	Yes, N = 30	20 (66.7%)	10 (33.3%)	19 (63.3%)	11 (36.7%)	12 (40%)	18 (60%)	8 (26.7%)	22 (73.3%)	20 (66.7%)	10 (33.3%)	11 (36.7%)	19 (63.3%)
	No, N = 104	37 (35.6%)	67 (64.4%)	47 (45.2%)	57 (54.8%)	34 (32.7%)	70 (67.3%)	32 (30.8%)	72 (69.2%)	48 (46.2%)	56 (53.8%)	2 (1.9%)	102 (98.1%)
*p-value*		0.002 *		0.079 *		0.457 *		0.665 *		0.047 *		< 0.001 Ω	
Extra-prostatic extension, number of cases (%)	Yes, N = 59	27 (45.8%)	32 (54.2%)	32 (54.2%)	27 (45.8%)	21 (35.6%)	38 (64.4%)	22 (37.3%)	37 (62.7%)	34 (57.6%)	25 (42.4%)	8 (13.6%)	51 (86.4%)
	No, N = 75	30 (40%)	45 (60%)	34 (45.3%)	41 (54.7%)	25 (33.3%)	50 (66.7%)	18 (24%)	57 (76%)	34 (45.3%)	41 (54.7%)	5 (6.7%)	70 (93.3%)
*p-value*		0.502 *		0.306 *		0.784 *		0.095 *		0.157 *		0.181 *	
Seminal vesicle invasion, number of cases (%**)**	Yes, N = 7	4 (57.1%)	3 (42.9%)	6 (85.7%)	1 (14.3%)	4 (57.1%)	3 (42.9%)	1 (14.3%)	6 (85.7%)	5 (71.4%)	2 (28.6%)	3 (42.9%)	4 (57.1%)
	No, N = 125	52 (41.6%)	73 (58.4%)	59 (47.2%)	66 (52.8%)	41 (32.8%)	84 (67.2%)	39 (31.2%)	86 (68.8%)	61 (48.8%)	64 (51.2%)	10 (8%)	115 (92%)
*p-value*		0.457 Ω		0.060 Ω		0.229 Ω		0.675 Ω		0.440 Ω		0.021 Ω	
Resection margin status, number of cases (%)	Affected, N = 60	27 (47.4%)	33 (42.9%)	33 (50%)	27 (39.7%)	21 (45.6%)	39 (44.3%)	18 (45%)	42 (44.7%)	31 (45.6%)	29 (43.9%)	6 (46.2%)	54 (44.6%)
	Unaffected, N = 74	30 (52.6%)	44 (47.1%)	33 (50%)	41 (60.3%)	25 (54.4%)	49 (55.7%)	22 (55%)	52 (55.3%)	37 (54.4%)	37 (56.1%)	7 (53.8%)	67 (55.4%)
*p-value*		0.603 *		0.230 *		0.882 *		0.974 *		0.847 *		0.916 *	

*P*-values are obtained from ^Ψ^ Wilcoxon Mann Whitney, * Pearson Chi Square or ^Ω^ Fisher’s Exact tests

The impact of these alterations on PSA recurrence according to PCa focality was analyzed. In the subgroup of UF tumors, only SLC45A3 *wt* expression showed an association with a shorter time to PSA recurrence (HR 0.13, *p* = 0.052) (Fig. 1b). In the subgroup of MF tumors, SPOP loss (HR 2.26, *p* = 0.043) (Fig. 1c) and the “triple hit” phenotype (HR 2.78, *p* = 0.041) (Fig. 1d) also showed an association with a shorter time to PSA recurrence. Moreover, a multivariate COX proportional hazard regression analysis was performed to elucidate if the association between expression alterations and a shorter time to PSA recurrence in UF or MF PCa was maintained when other clinicopathological characteristics were considered (Supplementary Table 2). The results indicated that the correlation between PSA recurrence and SLC45A3 *wt* in UF cases was close to be preserved (HR 7.83, *p* = 0.067). By contrast, the relationship between PSA recurrence and SPOP loss (HR 1.55, *p* = 0.344) or the “triple hit” phenotype (HR 1.34, *p* = 0.647) in MF tumors was lost.

### Immunohistochemical expression in tumor foci with different aggressiveness

**Fig. 2:**
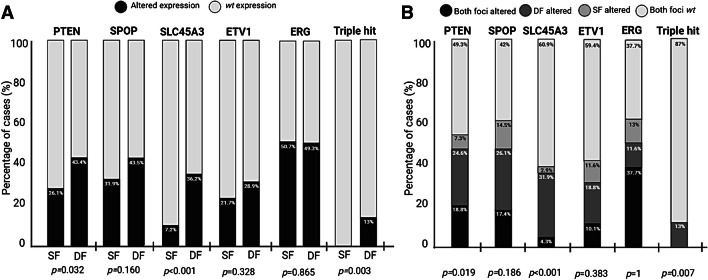
Immunohistochemical expression in tumor foci with different aggressiveness. *P*-values are obtained from Pearson Chi-Square tests (**a**). Immunohistochemical heterogeneity between paired DF and SF from MF PCa. *P*-values are obtained from McNemar tests (**b**). DF: dominant focus, SF: secondary focus

In a subgroup of 69 MF PCa, the immunohistochemical expression was analyzed in both the dominant focus (DF, with the highest GG) and a secondary focus (SF, a second focus with lower GG) (Fig. 2a). Considering PTEN status in the overall number of foci (69 DF and 69 SF), expression loss was statistically associated with the DF, as it was detected in 43.4% of DF but in only 26.1% of SF (*p* = 0.032). There was a statistical association between SLC45A3 expression loss and the DF, as it was found in 36.2% of DF but in only 7.2% of SF (*p* < 0.001). Regarding SPOP, expression loss was more frequently detected in DF (43.5%) rather than in SF (31.9%), but there were no significant differences. For ETV1 and ERG, both foci showed similar percentages of overexpression. Finally, the “triple hit” phenotype was detected in 13% of the DF, but in none of the SF (*p* = 0.003).

### Tumor foci heterogeneity in multifocal prostate cancer

The heterogeneity between paired DF and SF from the same 69 MF PCa was assessed (Fig. 2b). Homogeneity or heterogeneity were established when both foci showed concordant or discordant immunostaining patterns, respectively. Comparing the paired foci one by one, PTEN expression showed significant heterogeneity (*p* = 0.019), and the DF were more frequently altered than the SF (24.6% *vs* 7.3%). In 18.8% of cases both foci displayed homogeneous PTEN loss. SPOP homogeneous loss was detected in 17.4% of cases, while loss exclusively in the DF or in the SF was found in 26.1% and in 14.5%, respectively (*p* = 0.186). Data for SLC45A3 indicated high heterogeneity (*p* < 0.001), as homogeneous loss in both foci was very uncommon (4.3%). Among the cases with heterogeneous loss, most of the alterations were found in the DF (31.9%), and only 2.9% exhibited loss exclusively in the SF. ETV1 overexpression was homogeneously found in 10.1% of cases, while exclusively detected in the DF from 18.8%, and in the SF from 11.6% cases (*p* = 0.383). On the contrary, ERG expression showed the highest homogeneity (*p* = 1), given that 37.7% of cases overexpressed ERG in both foci, and also 37.7% were absolutely *wt*. No focus predominated among the discordant cases (11.6% *vs* 13%). Finally, all cases with the “triple hit” phenotype showed a heterogeneous pattern, as it was exclusively detected in their DF (*p* = 0.007).

## Discussion

The finding of multiple tumor foci in different anatomical zones of the prostate is particularly frequent [1, 5–8, 10], but multifocality is not an exclusive feature of PCa [25–27]. The impact of tumor multifocality in PCa prognosis has been addressed in several studies with conflicting results [5, 16]. Some authors have reported that MF and UF prostatic carcinomas may be biologically different, with the former being more aggressive, less differentiated and showing higher stage and shorter time to PSA recurrence [28], and even that some of the secondary foci in a patient with MF disease may have clinical significance [4, 6–8, 29, 30]. Nevertheless, in another study patients with UF tumors showed higher rates of positive surgical margins, high Gleason score and shorter time to PSA recurrence [31]. Other studies have failed to demonstrate any relationship between tumor focality and PCa clinicopathological features [7, 32, 33].

Our data from a series of PCa distributed in UF and MF cases, suggested a lack of association between tumor focality and most of the clinicopathological characteristics assessed, including tumor stage, PSA recurrence, perineural infiltration, extra-prostatic extension, seminal vesicle invasion, and resection margin status. Our findings clearly indicated that patients with MF PCa are diagnosed earlier than the ones with UF disease. Despite observing a significantly different case distribution in UF and MF PCa according to the GG, there was no consistent trend towards more aggressiveness for neither of them.

Numerous studies have described alterations in genes belonging to the two major carcinogenic pathways for PCa [19, 34–36], such as *PTEN*, *SPOP*, *SLC45A3*, *ETV1* and *ERG*. In this regard, our group reported significant associations between alterations in the expression of these proteins and prognosis [21–24, 37]. Nevertheless, the role of alterations in these genes on tumor focality and inter-foci heterogeneity in MF disease is still an area deserving further research. In the present study, immunostaining for PTEN, SPOP, SLC45A3, ETV1 and ERG was evaluated in a large and well characterized cohort of UF and MF PCa. Interestingly, only ETV1 overexpression was associated with PCa focality, as it was more frequently altered in UF cases. This is in agreement with our previous research in a different cohort of PCa already pointing at this association [22]. Despite PTEN loss was found at a higher incidence in MF cases, the association was not statistically significant. UF and MF tumors showed similar SPOP, SLC45A3, ERG and “triple hit” expression patterns.

Regarding clinicopathological characteristics and tumor focality, ERG overexpression showed a trend to be associated with a younger age in both MF and UF cases. This finding is in concordance with other studies suggesting a relationship between ERG overexpression and young-age patients, but they did not take into account PCa focality [38–40]. Lack of alterations in SLC45A3, SPOP, ETV1 or in the “triple hit”, were related to low GG tumors exclusively in MF cases. In line with these results, alterations in the expression of PTEN, SPOP, ERG or the “triple hit” phenotype were associated with adverse clinicopathological features in MF PCa, including high tumor stage, perineural infiltration, seminal vesicle invasion or PSA recurrence. By contrast, in UF PCa, only the overexpression of ETV1 and ERG were related to high tumor stage and perineural infiltration, respectively. It is worth noting that SLC45A3 *wt* was associated with PSA recurrence. Therefore, alternative molecular driver alterations may be characterizing this subset of SLC45A3 *wt* UF PCa.

In a subgroup of MF PCa, immunohistochemical expression was analyzed in both the dominant and the secondary foci. Taking into account the results from the overall number of foci, PTEN and SLC45A3 expression loss, as well as the “triple hit” phenotype, were statistically associated with the DF. Previous studies already suggested that low- and high-grade PCa foci may exhibit distinct expression signatures, with an enrichment of alterations in high GG foci [13, 15]. The results from the McNemar test, comparing the paired foci from MF cases, confirmed that PTEN and SLC45A3 immunostaining patterns, as well as the “triple hit” phenotype, were highly heterogeneous in our series. On the contrary, ERG showed highly concordant immunohistochemical expression in both foci. Altogether, these data agree with previous studies reporting that ERG overexpression is less heterogeneous in MF disease, while *PTEN* loss consistently exhibits variable expression patterns [41, 42]. In this regard, *TMPRSS2-ERG* fusion has been considered as an initial event in PCa [24, 37, 43–45], and this could be the reason for the high homogeneity in ERG immunostaining. Conversely, PTEN loss and *SLC45A3-ERG* fusion have been defined as more advanced and secondary events that take place after *TMPRSS2-ERG* fusion [21, 24, 41, 46–48]. Our finding of a heterogeneous expression pattern for PTEN and SLC45A3 would support this hypothesis.

In conclusion, our data support the hypothesis that UF and MF PCa may be different molecular entities. The study of an immunohistochemical panel, composed by PTEN, SPOP, SLC45A3, ETV1 and ERG, could provide prognostic information about the outcome of MF cases. Our findings will require prospective validation in a larger cohort of patients with MF PCa, and more research is needed to identify molecular alterations with prognostic value in the UF subgroup.

### Supplementary Information

Below is the link to the electronic supplementary material.

Supplementary file1 (JPG 1193 KB)

Supplementary file2 (DOCX 21 KB)

Supplementary file3 (DOCX 16 KB)

## Data Availability

The datasets used and/or analyzed during the current study are available from the corresponding author on reasonable request.
