# Supplementary figures and images for: Immunohistochemical markers as predictors of prognosis in multifocal prostate cancer
Source: Virchows Arch. 2023 Nov 28;485(2):281–90. doi: 10.1007/s00428-023-03699-z (PMC11329545; doi:10.1007/s00428-023-03699-z)

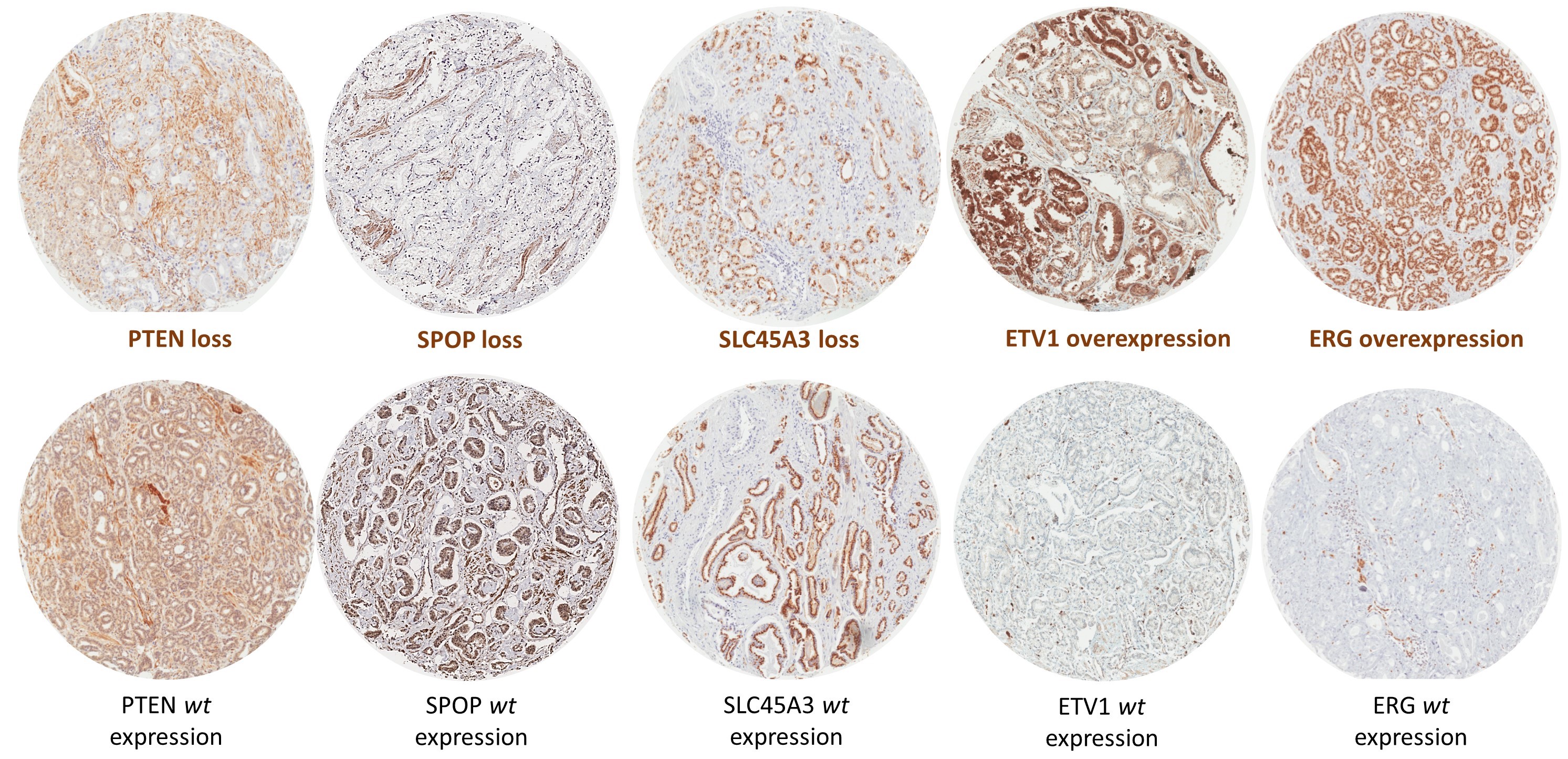

Supplement: Supplementary file 1 — Supplementary file1 (JPG 1193 KB) [file 428_2023_3699_MOESM1_ESM.jpg]
