# Supplementary material for: Immunohistochemical markers as predictors of prognosis in multifocal prostate cancer
Source: Virchows Arch. 2023 Nov 28;485(2):281–90. doi: 10.1007/s00428-023-03699-z (PMC11329545; doi:10.1007/s00428-023-03699-z)
Supplement: Supplementary file 2 — Supplementary file2 (DOCX 21 KB) [file 428_2023_3699_MOESM2_ESM.docx]

| **UNIFOCAL PCa**  **N=51** | | **PTEN** | | | **SPOP** | | | **SLC45A3** | | | **ETV1** | | | **ERG** | | | **Triple hit** | | |
| --- | --- | --- | --- | --- | --- | --- | --- | --- | --- | --- | --- | --- | --- | --- | --- | --- | --- | --- | --- |
|  |  | **loss** N=16 | ***wt*** N=35 | | **loss** N=27 | | ***wt*** N=24 | **loss** N=15 | ***wt*** N=36 | | **over- expression** N=24 | | ***wt*** N=27 | **over- expression** N=27 | | ***wt*** N=24 | **Yes** N=5 | **No** N=46 | |
| **Age at diagnosis,** range (average) | | 54 to 74 (67.4) | 56 to 80 (66.7) | | 56 to 80 (66.7) | | 54 to 74 (67.1) | 57 to 74 (67.8) | 54 to 80 (66.5) | | 56 to 73 (67.7) | | 54 to 80 (66.2) | 54 to 74 (65.6) | | 54 to 80 (68.4) | 63 to 70 (65.8) | 54 to 80 (67) | |
| *p-value* | | 0.854 Ψ | | | 0.582 Ψ | | | 0.787 Ψ | | | 0.820 Ψ | | | 0.058 Ψ | | | 0.381 Ψ | | |
| **GG at diagnosis,** number of cases (%) | **GG1** N=8 | 0 (0%) | 8 (100%) | | 2 (25%) | | 6 (75%) | 0 (0%) | 8 (100%) | | 2 (25%) | | 6 (75%) | 4 (50%) | | 4 (50%) | 0 (0%) | 8 (100%) | |
|  | **GG2** N=15 | 4 (26.7%) | 11 (73.3%) | | 8 (53.3%) | | 7 (46.7%) | 3 (20%) | 12 (80%) | | 6 (40%) | | 9 (60%) | 7 (46.7%) | | 8 (53.3%) | 0 (0%) | 15 (100%) | |
|  | **GG3** N=12 | 5 (41.7%) | 7 (58.3%) | | 7 (58.3%) | | 5 (41.7%) | 4 (33.3%) | 8 (66.7%) | | 7 (58.3%) | | 5 (41.7%) | 6 (50%) | | 6 (50%) | 1 (8.3%) | 11 (91.7%) | |
|  | **GG4** N=5 | 1 (20%) | 4 (80%) | | 3 (60%) | | 2 (40%) | 2 (40%) | 3 (60%) | | 3 (60%) | | 2 (40%) | 2 (40%) | | 3 (60%) | 1 (20%) | 4 (80%) | |
|  | **GG5** N=11 | 6 (54.5%) | 5 (45.5%) | | 7 (63.6%) | | 4 (36.4%) | 6 (54.5%) | 5 (45.5%) | | 6 (54.5%) | | 5 (45.5%) | 8 (72.7%) | | 3 (27.3%) | 3 (27.3%) | 8 (72.7%) | |
| *p-value* | | 0.102 Ω | | | 0.547 Ω | | | 0.086 Ω | | | 0.567 Ω | | | 0.696 Ω | | | 0.094 Ω | | |
| **Tumor stage (pT),** number of cases (%) | **pT2** N=37 | 12 (32.4%) | 25 (67.6%) | | 21 (56.7%) | | 16 (43.3%) | 10 (27%) | 27 (73%) | | 14 (37.8%) | | 23 (62.2%) | 19 (51.5%) | | 18 (48.5%) | 3 (8.1%) | 34 (91.9%) | |
|  | **pT3** N=14 | 4 (28.6%) | 10 (71.4%) | | 6 (42.9%) | | 8 (57.1%) | 5 (35.7%) | 9 (64.3%) | | 10 (71.4%) | | 4 (28.6%) | 8 (57.1%) | | 6 (42.9%) | 2 (14.3%) | 12 (85.7%) | |
| *p-value* | | 1.000 Ω | | | 0.375 * | | | 0.732 Ω | | | **0.031** * | | | 0.711 * | | | 0.606 Ω | | |
| **Perineurial infiltration,** number of cases (%) | **Yes** N=13 | 6 (46.1%) | 7 (53.9%) | | 6 (46.1%) | | 7 (53.9%) | 5 (38.5%) | 8 (61.5%) | | 9 (69.2%) | | 4 (30.8%) | 10 (76.9%) | | 3 (23.1%) | 2 (15.4%) | 11 (84.6%) | |
|  | **No** N=38 | 10 (26.3%) | 28 (73.7%) | | 21 (55.3%) | | 17 (44.7%) | 10 (26.3%) | 28 (73.7%) | | 15 (39.5%) | | 23 (60.5%) | 17 (44.7%) | | 21 (55.3%) | 3 (7.9%) | 35 (92.1%) | |
| *p-value* | | 0.298 Ω | | | 0.806 Ω | | | 0.487 Ω | | | 0.063 * | | | **0.044** * | | | 0.591 Ω | | |
| **Extra-prostatic extension,** number of cases (%) | **Yes** N=27 | 11 (40.7%) | 16 (59.3%) | | 13 (48.1%) | | 14 (51.9%) | 10 (37%) | 17 (63%) | | 15 (55.5%) | | 12 (44.5%) | 17 (63%) | | 10 (37%) | 3 (11.1%) | 24 (88.9%) | |
|  | **No** N=24 | 5 (20.8%) | 19 (79.2%) | | 14 (58.3%) | | 10 (41.7%) | 5 (20.8%) | 19 (79.2%) | | 9 (37.5%) | | 15 (62.5%) | 10 (41.7%) | | 14 (58.3%) | 2 (8.3%) | 22 (91.7%) | |
| *p-value* | | 0.126 * | | | 0.467 * | | | 0.205 * | | | 0.197 * | | | 0.128 * | | | 1.000 Ω | | |
| **Seminal vesicle invasion,** number of cases (%) | **Yes** N=5 | 1 (20%) | 4 (80%) | | 1 (20%) | | 4 (80%) | 2 (40%) | 3 (60%) | | 4 (80%) | | 1 (20%) | 3 (60%) | | 2 (40%) | 1 (20%) | 4 (80%) | |
|  | **No** N=46 | 15 (32.6%) | 31 (67.4%) | | 26 (56.5%) | | 20 (43.5%) | 13 (28.3%) | 33 (71.7%) | | 20 (43.5%) | | 26 (56.5%) | 24 (52.2%) | | 22 (47.8%) | 4 (8.7%) | 42 (91.3%) | |
| *p-value* | | 1.000 Ω | | | 0.175 Ω | | | 0.624 Ω | | | 0.175 Ω | | | 1.000 Ω | | | 0.416 Ω | | |
| **Resection margin status,** number of cases (%) | **Affected**  N=23 | 7  (43.8%) | | 16  (45.7%) | 13  (48.1%) | 10  (41.7%) | | 7  (46.7%) | | 16  (44.4%) | 9  (37.5%) | 14  (51.9%) | | 13  (48.1%) | 10  (41.7%) | | 2  (40%) | | 21  (45.7%) |
|  | **Unaffected**  N=28 | 9  (56.2%) | | 19  (54.3%) | 14  (51.9%) | 14  (58.3%) | | 8  (53.3%) | | 20  (55.6%) | 15  (62.5%) | 13  (48.1%) | | 14  (51.9%) | 14  (58.3%) | | 3  (60%) | | 25  (54.3%) |
| *p-value* | | 0.896 * | | | 0.642 * | | | 0.884 * | | | 0.304 * | | | 0.642 * | | | 1.000 Ω | | |
| *P*-values are obtained from ^Ψ^ Wilcoxon Mann Whitney, * Pearson Chi Square or ^Ω^ Fisher’s Exact tests. | | | | | | | | | | | | | | | | | | | |
