# Supplementary material for: Immunohistochemical markers as predictors of prognosis in multifocal prostate cancer
Source: Virchows Arch. 2023 Nov 28;485(2):281–90. doi: 10.1007/s00428-023-03699-z (PMC11329545; doi:10.1007/s00428-023-03699-z)
Supplement: Supplementary file 3 — Supplementary file3 (DOCX 16 KB) [file 428_2023_3699_MOESM3_ESM.docx]

| **Variable (UF)** | **HR** | **Lower CI** | **Upper CI** | ***p-value*** |
| --- | --- | --- | --- | --- |
| **SLC45A3 *wt*** | 7.837 | 0.864 | 71.122 | 0.067 |
| **Age at diagnosis** | 1.039 | 0.946 | 1.142 | 0.424 |
| **GG2** | 0.733 | 0.177 | 3.042 | 0.669 |
| **GG3** | 0.577 | 0.056 | 5.881 | 0.642 |
| **GG4** | 3.355 | 0.455 | 24.759 | 0.235 |
| **GG5** | 1.291 | 0.190 | 8.746 | 0.794 |
| **Tumor stage (pT)** | 0.168 | 0.015 | 1.863 | 0.146 |
| **Perineurial infiltration** | 7.811 | 1.038 | 58.752 | **0.046** |
| **Extra-prostatic extension** | 0.205 | 0.033 | 1.260 | 0.087 |
| **Seminal vesicle invasion** | 0.828 | 0.036 | 18.889 | 0.906 |
| **Resection margin status** | 3.992 | 0.982 | 16.225 | **0.053** |
| **Variable (MF)** | **HR** | **Lower CI** | **Upper CI** | ***p-value*** |
| **SPOP loss** | 1.554 | 0.623 | 3.875 | 0.344 |
| **Age at diagnosis** | 1.063 | 0.993 | 1.139 | 0.080 |
| **GG2+GG3** | 1.746 | 0.446 | 6.841 | 0.423 |
| **GG4** | 1.865 | 0.359 | 9.701 | 0.458 |
| **GG5** | 1.103 | 0.254 | 4.794 | 0.896 |
| **Tumor stage (pT)** | 1.394 | 0.323 | 6.020 | 0.656 |
| **Perineurial infiltration** | 1.843 | 0.472 | 7.193 | 0.379 |
| **Extra-prostatic extension** | 0.378 | 0.130 | 1.096 | 0.073 |
| **Seminal vesicle invasion** | 10.950 | 2.979 | 40.244 | **<0.001** |
| **Resection margin status** | 4.503 | 1.747 | 11.611 | **0.002** |
| **Variable (MF)** | **HR** | **Lower CI** | **Upper CI** | ***p-value*** |
| **Triple hit** | 1.346 | 0.377 | 4.814 | 0.647 |
| **Age at diagnosis** | 1.061 | 0.989 | 1.138 | 0.097 |
| **GG2+GG3** | 1.787 | 0.461 | 6.928 | 0.401 |
| **GG4** | 1.945 | 0.375 | 10.087 | 0.428 |
| **GG5** | 1.173 | 0.271 | 5.082 | 0.831 |
| **Tumor stage (pT)** | 1.600 | 0.389 | 6.583 | 0.514 |
| **Perineurial infiltration** | 1.609 | 0.395 | 6.553 | 0.506 |
| **Extra-prostatic extension** | 0.404 | 0.137 | 1.191 | 0.100 |
| **Seminal vesicle invasion** | 10.932 | 2.983 | 40.063 | **<0.001** |
| **Resection margin status** | 4.439 | 1.746 | 11.285 | **0.002** |
